# Mouse "xenotropic" gammaretroviruses, XMRV and their XPR1 receptor

**DOI:** 10.1186/1742-4690-8-S2-O17

**Published:** 2011-10-03

**Authors:** Surendranath Baliji, Yuhe Yan, Yoshiaki Nishimura, Qingping Liu, Carrie Martin, Bernard Lafont, Christine A Kozak

**Affiliations:** 1 Laboratory of Molecular Microbiology, National Institute of Allergy and Infectious Diseases, Bethesda, MD, 20892-0460, USA

## Background

Xenotropic/polytropic mouse leukemia viruses (XP-MLVs) and the xenotropic murine leukemia virus-related virus (XMRV) identified in human patient samples rely on the XPR1 receptor for entry into cells. There are 5 functional variants of *Xpr1* in *Mus* species and laboratory mouse strains that differ in their ability to support entry of XMRV and various isolates of XP-MLVs. Additional receptor variants are found in non-rodent vertebrate species.

## Materials and methods

Various vertebrate species were examined for XPR1 sequence differences and for differences in susceptibility to viral pseudotypes carrying the β-galactosidase reporter gene and 5 different virus *env* glycoproteins: XMRV, prototypical xenotropic and polytropic MLVs, and two wild mouse isolates with atypical host range. Novel receptor variants and mutants were cloned into expression vectors, expressed in nonpermissive hamster cells and evaluated for expression, receptor function and virus envelope binding. *In vivo* infectivity and pathogenic potential of X-MLV and XMRV were evaluated in rhesus macaque and in laboratory mice congenic for permissive receptor variants.

## Results

The species distribution of XP-MLVs and *Xpr1* variants in wild mouse populations indicates that restrictive receptor variants evolved in house mouse populations exposed to XP-MLV infection. Most primate species have the same permissive XPR1, but in domesticated birds, XPR1 receptor function varies due to substitutions at 2 sites that correspond to sites that are critical for X-MLV entry in mice (K500, T582). Substitutions at these 2 sites in mouse and bird *Xpr1* produce functionally distinct receptors, and evaluation of double mutants in the avian receptor suggests that these sites, in two extracellular loops, contribute to a single receptor determinant. XPR1 function was also examined in cells expressing 2 receptors with different infectivity profiles produced by cotransfection or by interspecific crosses.

Because of the initial suggestion that XMRV is linked to human disease, and the discovery that all wild mice and some laboratory strains are susceptible to X-MLV, we examined the ability of these viruses to establish acute infection and disease in mice carrying permissive XPR1 alleles and in macaques transiently immunosuppressed with anti-CD8 and anti-CD20 mAb. Macaque infection produced only a small transient plasma viremia. No alterations in disease profile or latency period were detected in naturally high leukemic AKR mice congenic for 2 different *Xpr1* alleles that were neonatally infected with X-MLV.

## Conclusions

Analysis of the species and geographical distribution of *Mus Xpr1* provides a good example of how genetic conflicts can drive diversifying selection. Battleground regions in receptor determining regions of XPR1 are marked by mutational changes that have produced substantive phenotypic changes in house mouse species exposed to virus infection, and mutational changes at the same sites alter receptor function in domesticated avian species. Although these changes suggest positive selection favors antiviral alleles in species exposed to XP-MLV, inoculations of permissive laboratory mice and immunosuppressed macaques suggest that host factors effectively limit virus spread and disease induction.

